# Effects of Long-Term Periodic Submergence on Photosynthesis and Growth of *Taxodium distichum* and *Taxodium ascendens* Saplings in the Hydro-Fluctuation Zone of the Three Gorges Reservoir of China

**DOI:** 10.1371/journal.pone.0162867

**Published:** 2016-09-12

**Authors:** Chaoying Wang, Changxiao Li, Hong Wei, Yingzan Xie, Wenjiao Han

**Affiliations:** Key Laboratory of Eco-Environments in the Three Gorges Reservoir Region (Ministry of Education), Chongqing Key Laboratory of Plant Ecology and Resources in the Three Gorges Reservoir Region, School of Life Sciences, Southwest University, Chongqing, 400715, PR China; Agriculture and Agri-Food Canada, CANADA

## Abstract

Responses of bald cypress (*Taxodium distichum*) and pond cypress (*Taxodium ascendens*) saplings in photosynthesis and growth to long-term periodic submergence *in situ* in the hydro-fluctuation zone of the Three Gorges Dam Reservoir (TGDR) were studied. Water treatments of periodic deep submergence (DS) and moderate submergence (MS) *in situ* were imposed on 2-year-old bald cypress and pond cypress saplings. The effects of periodic submergence on photosynthesis and growth were investigated after 3 years (i.e. 3 cycles) compared to a control (i.e. shallow submergence, abbreviated as SS). Results showed that pond cypress had no significant change in net photosynthetic rate (*P*_*n*_) in response to periodic moderate and deep submergence in contrast to a significant decrease in *P*_*n*_ of bald cypress under both submergence treatments, when compared to that of SS. Ratios of Chlorophyll a/b and Chlorophylls/Carotenoid of pond cypress were significantly increased in periodic moderate submergence and deep submergence, while bald cypress showed no significant change. Diameter at breast height (DBH) and tree height of both species were significantly reduced along with submergence depth. Relative diameter and height growth rates of the two species were also reduced under deeper submergence. Moreover, bald cypress displayed higher relative diameter growth rate than pond cypress under deep submergence mainly attributed to higher productivity of the larger crown area of bald cypress. When subjected to deep subergence, both species showed significant reduction in primary branch number, while in moderate submergence, bald cypress but not pond cypress showed significant reduction in primary branch number. These results indicate that both bald cypress and pond cypress are suitbale candidates for reforestation in the TGDR region thanks to their submergence tolerance characteristics, but bald cypress can grow better than pond cypress under deep submergence overall.

## Introduction

Dam building has altered the natural hydrological regimes of many rivers [1,2] all over the world. The Three Gorges Dam in the upper reaches of the Yangtze River, as the largest dam ever built in China [3] for the purposes of flood control, hydropower generation and navigation, has disrupted the natural dynamic equilibrium of free-flowing river systems [4] and created a huge reservoir. To operate the TGDR at full capacity, the water level of the TGDR fluctuates between 145 m a.s.l. in summer (i.e. from May to September mainly for flood control and emission sediment) and 175 m a.s.l. in winter (i.e. from October to the following April mainly for energy generation and navigation) [5,6]. These extreme fluctuations with an annual 30 m water-level drawdown have led to the formation of a hydro-fluctuation zone around the reservoir. The newly-formed hydro-fluctuation zone in the TGDR encompasses an area of 400 km² and 2000 km of shoreline [7]. This hydro-fluctuation zone now experiences a reversed flooding seasonality and prolonged flooding duration [8], which is opposite to its natural hydrological regime of the Yangtze River [6]. In the hydro-fluctuation zone, annual submergence stress can last as long as 182 days at 165 m a.s.l. and 364 days at 146 m a.s.l. However, the implications of such hydrological operations for the survival and growth of woody plants growing in the upper elevations of the hydro-fluctuation zone of the TGDR are not well known. There is thus a clear need to screen suitable tree species for vegetative restoration of the hydro-fluctuation zone of the TGDR [9].

Since its first impoundment in 2003, the operation of the artificial flow regime has caused serious degradation of the vegetation in the hydro-fluctuation zone of the TGDR due to plants’ intolerances to such off-season submergence [8], which directly led to a decline in riparian ecosystem structure and functioning. Vegetation is the most important functional agent by maintaining biological productivity and biodiversity of the hydro-fluctuation zone [10] of the TGDR. Revegetation is an environmentally friendly measure in restoring ecological integrity of the hydro-fluctuation zone [11,12] of the TGDR, and also crucial for maintaining sound ecosystem functions and services within the newly established riparian ecosystems. However, it is a great challenge to successfully revegetate the degraded hydro-fluctuation zone because of the tolerance plants need to have to such extreme environmental adversity [13].

*Taxodium distichum* L. Rich and *Taxodium ascendens* Brongn., two deciduous tree species native to southeastern North America, have become widespread throughout the Yangtze River valleys of China since they were introduced 85 years ago [7]. As an ancient lineage of conifers that are well-adapted to hydric habitats, both are bottomland species with water tolerance [14–21] and drought endurance [9,21], and therefore promising candidates for revegetation of the hydro-fluctuation zone of the TGDR [9]. Bald cypress with more linear leaves, usually grows in nutrient-rich flood plains of large rivers, whereas, pond cypress, being characterized by sclerophyllous awl-like leaves, grows in nutrient-poor riparian zones of bays and creeks. Both species can produce pneumatophores to survive and grow in flooded habitats. Although a number of former studies documented the photosynthetic response [17,20,22–24], growth [14,15,19,25], as well as metabolic and physiochemical change [7] of bald cypress and pond cypress under a range of hydrological regimes, most of the studies were based on controlled garden experiments and little research has been conducted *in situ* in the hydro-fluctuation zone of the TGDR [8], leaving question-marks about the applicability of both species for vegetative restoration of the hydro-fluctuation zone of the TGDR. In fact, the results obtained from the controlled experimental garden studies have often been inconsistent with the outcomes of *in situ* field application, and thus of limited use to guide restoration of the riparian forest of the TGDR [8].

Moreover, those garden experiments mostly used seedlings of bald cypress and pond cypress [20,26] and imposed shallow [20,27] and short-term [7,21,27] submergence treatment, even though the seedlings may not survive and grow well *in situ* due to strong intensity and long duration of off-season submergence in the hydro-fluctuation zone of the TGDR. Considering the response differences between seedlings and saplings to water depth and duration of submergence stress [9,28,29], two-year-old bald cypress and pond cypress saplings might be able to recover from long-term and deep periodic submergence when planted in the hydro-fluctuation zone of the TGDR. In addition, among the studies cited, only Li et al. [7] compared their tolerant abilities under simulated hydrological regimes and found that pond cypress was more resilient than bald cypress due to higher root malate and shikimate content and leaf photosynthesis. In order to clarify the resistance of the two species to the altered hydrological regimes *in situ* of the TGDR, comparative field study is still needed. The main objective of this study was to quantify the photosynthentic and growth responses of bald cypress and pond cypress saplings to continuous 3-year cycles of water level change of the TGDR and provide tools for revegetation of the hydro-fluctuation zone of the TGDR. Thus, we hypothesized that: 1) the photosynthesis and growth of the two species will decline along with lower elevation due to longer duration of submergence stress; 2) slow growth rate of the plants at lower elevation has a significant relationship with photosynthesis down regulation in the context of the hydro-fluctuation zone of the TGDR; 3) pond cypress will be more resilient when applied to vegetation restoration of the hydro-fluctuation zone of the TGDR region.

## Materials and Methods

### Study Site and Experimental Materials

The study site (30°24′16″ ~ 30°24′56″ N, 108°08′03″ ~ 108°08′21″ E) is located in Ruxi River basin in Gonghe Village of Shibao Township, Zhong County, Chongqing municipality of China (about 32 km distance to Zhong County). Ruxi River is one of the largest tributaries of the TGDR [30]. The basin is characterized by subtropical southeast monsoonal climate, with average annual temperature of 18.2°C and 1327.5 h of sunshine per year. Annual precipitation is 1200 mm and relative humidity is 80%. The site has purple soil, formed in the parent material of calcareous purple sand shale typical for the Chinese subtropics. The weathering of rock is shallow, and the soil maturation degree is low. This has led to serious soil and water erosion in the less-vegetated riparian zone of the Ruxi River.

**Fig 1:**
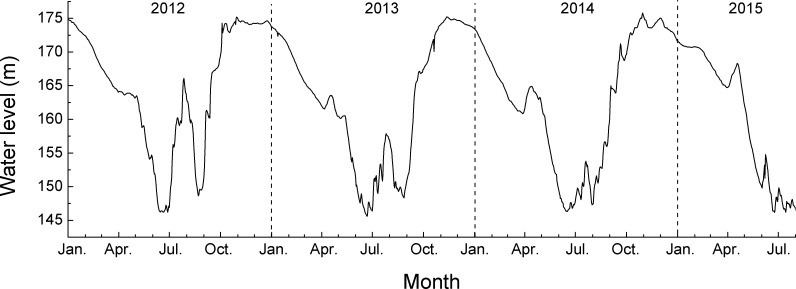
Water level change of the hydro-fluctuation zone of the Three Gorges Dam Reservoir in Zhong County from January 2012 through July 2015.

To conduct *in situ* experimentation to rehabilitate the vegetation of the hydro-fluctuation zone of the Three Gorges Reservoir, our research obtained a permission from Chongqing Forestry Department and started artificial vegetation construction through reforestation with two-year-old bald cypress and pond cypress saplings in this site in April 2012. Two-year old saplings of bald cypress and pond cypress were purchased from the Dajuyuan Tree Seedling Nursery in Chongqing municipality. The baseline data measured on those saplings prior to planting are in Table 1. Saplings of both bald cypress and pond cypress were immediately planted at the upper portion of the hydro-fluctuation zone of Ruxi River (i.e. the water-level fluctuation belt between 165 m a.s.l. and 175 m a.s.l.) after transport from the nursery. For preparation of the reforestation, the upper portion was first divided into twenty plots of 10 meters width each, perpendicular to the river-bed. Both species were planted in an alternating pattern among these plots with a spacing of 1 m × 1 m, while one plot contained only one species. Trees were well watered once immediately after being planted, and then weeded in mid June of 2012. Then, no watering or weeding was conducted anymore in the following years. The survival rate of the planted bald cypress and pond cypress saplings was 100%. Until measurement, these saplings had grown well and had already experienced three cycles of water level fluctuation imposed by the TGDR. The water level of the hydro-fluctuation zone of the TGDR in Zhong County where the research site is located, was gradually raised from September through October, and maintained at the highest water level of 175 m a.s.l. from November through Juanary, to be followed by gradual drawdown until reaching its lowest water level of 145 m a.s.l. from June to September (see Fig 1).

**Table 1:** Baseline data on tree height and diameter at breast height (DBH) of *T*. *distichum* and *T*. *ascendens* saplings prior to planting in 2012 (means ± S.E., n = 9).

Index	*T*. *distichum*	*T*. *ascendens*
DBH (cm)	0.76 ± 0.06a^†^	0.78 ± 0.06a
Tree height (m)	1.61 ± 0.01a	1.64 ± 0.01a

^†^According to *t*-tests, the values did not differ significantly between species at the 0.05 level.

### Experimental Design

The study site of the hydro-fluctuation zone of Ruxi River was divided into three elevations, 165 m, 170 m and 175 m a.s.l., which stand for the deep submergence (DS), moderate submergence (MS) and shallow submergence (SS, serving as control), respectively. Submergence depth and duration of saplings at each elevation is shown in Table 2. At each elevation, nine representative saplings per species, i.e. one in each plot (leaving one plot per species unused), were randomly selected for further testing. To avoid the effect of light and space on plant growth, trees located at the edge of the plantation belt were avoided.

**Table 2:** Submergence depth and duration of the treatments at different elevations during the three water cycles.

Elevation (m)	Submergence depth (m)^†^	Submergence duration (d)		
		From July 2012 to June 2013	From July 2013 to June 2014	From July 2014 to June 2015
165	10	175	158	217
170	5	125	101	141
175	0	2	5	8

^†^The submergence depth refers to the water depth above the soil surface in which saplings of bald cypress and pond cypress were planted. The submergence depth above the top of the saplings varied due to operation of the Three Gorges Dam Reservoir, and can be calculated as the difference between the submergence depth given in this table and the tree height.

### Measurement of Physiological Responses

All bald cypress and pond cypress saplings on the study site were identified and tagged on July 12, 2015 when in the middle of their 4^th^ growing season. Nine saplings for each species at each elevation (i.e. one per plot) were randomly chosen to test their responses of leaf gas exchange and leaf chlorophyll content. Gas exchange measurements were conducted using the Li-Cor LI-6400 Portable Operation System (Li-Cor, Inc., Lincoln, NE, USA) equipped with a standard 6 cm^2^ leaf chamber with an internal photoactive radiation sensor, after the saplings had been induced under saturated light illumination (red/blue light source) of 1000 μmol photons m^-2^ s^-1^. This photosynthetic photon flux density (PPFD) was chosen according to previous determination of the light-saturation curve, which indicated that saplings were light-saturated at this level. All of the tests were taken between 10:00 am– 15:00 pm on a fine day [7,31] with cuvette conditions: at a PPFD, 1000 μmol photons m^-2^ s^-1^; flow rate, 500 μmol s^-1^; vapour pressure deficit (VPD), 1.02 ± 0.03 kPa (mean ± SD); leaf temperature, 25°C. The third or fourth mature, intact compound, upper leaf located on a branch in the upper canopy was utilized for the measurements of net photosynthetic rate (*P*_*n*_), stomatal conductance (*g*_*s*_), intercellular CO_2_ concentration (*C*_*i*_) and ambient CO_2_ concentration (*C*_*a*_). After data recording, the leaf was labeled with a marker pen, and then rapidly picked and put between two sheets of wet filter paper to keep moist. The leaf in cooled condition was then taken to the laboratory to measure leaf area by WinRHIZO, LC4800-II LA2400. The intrinsic water use efficiency (WUEi) was determined by the ratio of *P*_*n*_ to *g*_*s*_ [9]. The limiting value of stomata (Ls) was calculated by the formula Ls = 1 –*C*_*i*_/*C*_*a*_ [32]. Leaf chlorophylls (Chls) and carotenoid (Car) content of each sapling was measured after extracting by 80% acetone in the dark for 72 h at 4°C. The absorbance of extracts was measured at 663, 646 and 470 nm with spectrophotometer UV/VIS 2550 (Shimadzu, Japan).

### Measurement of Plant Growth

Diameters at breast height (1.3 m from the ground) (DBH) of the bald cypress and pond cypress saplings were measured at two perpendicular directions using vernier calipers and the average value was used in further analysis. Tree height was measured using a standard meter pole (with an accuracy of 1.0 cm). Crown diameters were measured at two perpendicular directions using a measuring tape and the average value was used in further analysis. Crown area was calculated using the formula of area of a circle, i.e. (π) × (mean diameter/2)^2^. Numbers of primary branches (with branch length ≥ 10 cm) on the trunk of the two species were recorded. To verify the height and diameter growth of each sapling after 3-year cycles of periodic submergence, we calculated the relative height growth rate (RHGR) and the relative diameter growth rate (RDGR) [33] using the following two equations:

$$RHGR\;({year}^{-1})=\frac{1}{H}∙\frac{dH}{dt}\approx \frac{\ln H2015-\ln H2012}{3\;year}$$

$$RDGR\;({year}^{-1})=\frac{1}{D}∙\frac{dD}{dt}\approx \frac{\ln D2015-\ln D2012}{3\;year}$$

where *H* 2015, 2012 and *D* 2015, 2012 indicate the tree height and DBH of the saplings in 2015 and 2012, respectively.

### Data Analysis

All statistical analyses were performed using SPSS 16.0 and Microsoft 2007 software. Analyses of the data employed general linear model (GLM) procedures to determine any significant overall differences among treatments. The premises of normality, homoscedasticity, and sphericity had been verified earlier. An ANOVA (Analysis of Variance) followed by Tukey’s test was used to determine significant differences at the 0.05 level among individual treatments. Within each submergence treatment, comparisons of photosynthesis and growth of the two species were analyzed with paired *t*-test. Pearson’s correlations between photosynthetic parameters and growth were calculated.

## Results

### Gas Exchange Response

**Fig 2:**
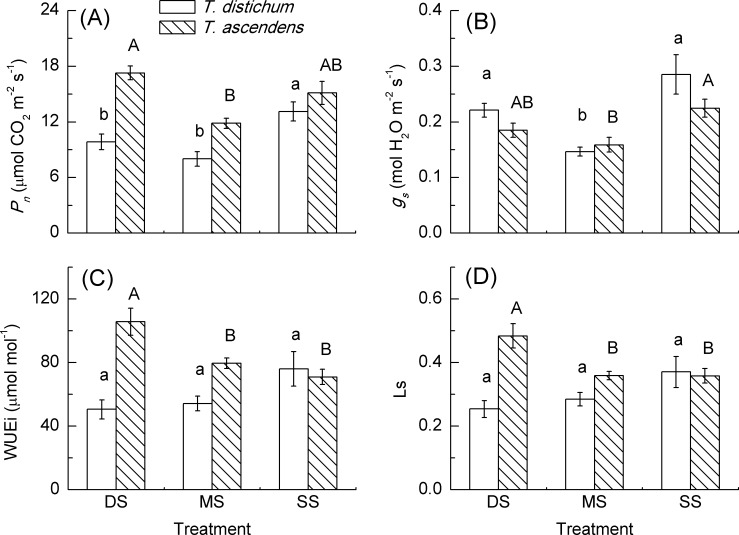
Comparisons of net photosynthetic rate (*P*_*n*_), stomatal conductance (*g*_s_), intrinsic water use efficiency (WUEi) and limiting value of stomata (Ls) of *T*. *distichum* (blank bars) and *T*. *ascendens* (diagonal bars) saplings under shallow submergence (SS), moderate submergence (MS) and deep submergence (DS). Values are means ± S.E. (n = 9). The values with different letters are significantly different at *P* < 0.05 according to Tukey’s test. Lower case letters are used for *T*. *distichum* saplings while upper case letters are used for *T*. *ascendens* saplings.

After experiencing three annual cycles of periodic submergence in the hydro-fluctuation zone of the TGDR, mean *P*_*n*_ of bald cypress in both MS and DS was significantly lower than that in SS, with a decrease of 39% and 25% respectively. There was no significant difference in *P*_*n*_ of bald cypress between MS and DS, whereas, mean *P*_*n*_ of pond cypress in DS was 46% higher than that of MS (*p* < 0.001). However, compared to mean *P*_*n*_ of pond cypress in SS, there was no significant difference with MS and DS, respectively (Fig 2A). No effect was demonstrated respectively for species and treatment (both *p* > 0.05) in *P*_*n*_, but there was an effect for species × treatment (*p* < 0.05) in *P*_*n*_. Pond cypress showed a higher *P*_*n*_ than bald cypress in MS and DS (MS: *p* < 0.01, DS: *p* < 0.001), respectively, but no difference in *P*_*n*_ was found between the two species in SS (*p* > 0.05).

Mean *g*_*s*_ in MS was significantly lower than that in SS, i.e. 49% lower in bald cpress and 29% lower in pond cypress, respectively. Compared to SS, no significant difference was exhibited in *g*_*s*_ of either bald cypress or pond cypress in DS (Fig 2B). Overall, there were no effects in *g*_*s*_ for species, treatment and species × treatment interaction (all *p* > 0.05). There was no difference in *g*_*s*_ between the two species at any elevation (all *p* > 0.05).

Mean WUEi of bald cypress showed no significant difference among three treatments. Mean WUEi of pond cypress in DS was significantly higher than that in both MS and SS, respectively, while WUEi of MS had no significant difference with that of SS in pond cypress (Fig 2C). Overall, there was no effect for species and treatment (both *p* > 0.05), but there was a species × treatment interaction (*p* < 0.01) in WUEi. Pond cypress showed a higher WUEi than bald cypress both in MS and DS (both *p* < 0.001), respectively, but no difference to be detected between the two species in SS (*p* > 0.05).

Mean Ls of bald cypress also had no significant difference among the three treatments. Mean Ls of pond cypress in DS was significantly higher than that in MS and SS, respectively. In contrast, no significant difference was displayed between MS and SS in Ls of pond cypress (Fig 2D). Overall, there were no significant effects for species, treatment and species × treatment (all *p* > 0.05) in Ls. Pond cypress showed a higher Ls than bald cypress in MS and DS, respectively (MS: *p* < 0.01, DS: *p* < 0.001), but there was no difference in Ls between the two species in SS (*p* > 0.05).

### Pigment Content in Leaves

**Fig 3:**
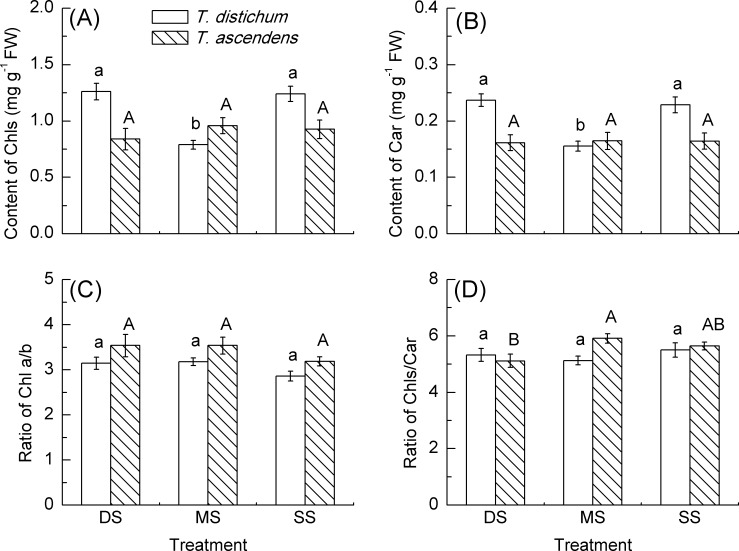
Comparisons of content of chlorophylls (Chls) and carotenoid (Car), ratio of chlorophyll (Chl) a/b and chlorophylls (Chls) / carotenoids (Car) of *T*. *distichum* (blank bars) and *T*. *ascendens* (diagonal bars) saplings under shallow submergence (SS), moderate submergence (MS) and deep submergence (DS). Values are means ± S.E. (n = 9). The values with different letters are significantly different at *P* < 0.05 according to Tukey’s test. Lower case letters are used for *T*. *distichum* saplings while upper case letters are used for *T*. *ascendens* saplings.

In bald cypress saplings, the Chls and Car contents in MS were significantly reduced by 36% and 32%, respectively, while no significant difference was found in DS, as compared to that in SS. In pond cypress, by contrast, the Chls and Car content in MS and DS had no significant difference with that in SS (Fig 3A and 3B). There was no significant difference in ratio of Chl a/b and ratio of Chls/Car in either species among the three treatments (Fig 3C and 3D).

Overall, there were effects for species, treatment and species × treatment (all *p* < 0.001) in Chls and Car content. In ratio of Chl a/b, there was an effect for species (*p* < 0.01), but no effect for treatment and species × treatment (both *p* > 0.05). In ratio of Chls/Car, there were no effects for species, treatment and species × treatment (all *p* > 0.05).

### Growth of Bald Cypress and Pond Cypress

**Fig 4:**
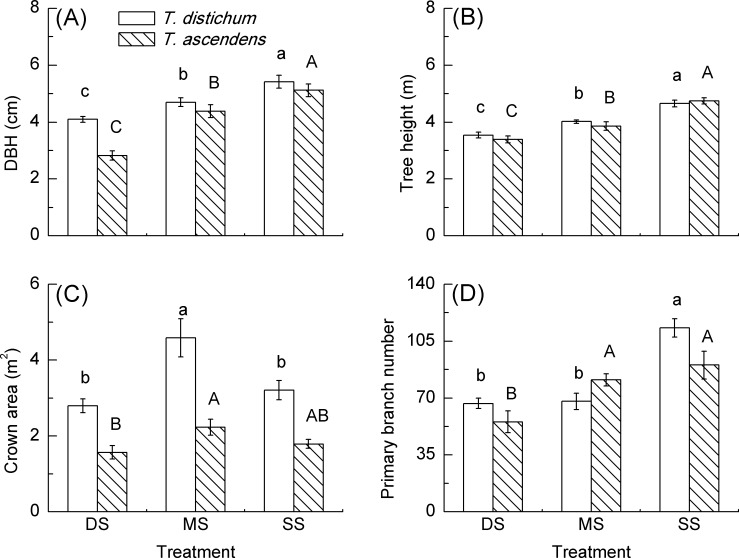
Comparisons of diameter at breast height (DBH), tree height, crown area and primary branch number of *T*. *distichum* (blank bars) and *T*. *ascendens* (diagonal bars) saplings under shallow submergence (SS), moderate submergence (MS) and deep submergence (DS). Values are means ± S.E. (n = 9). The values with different letters are significantly different at *P* < 0.05 according to Tukey’s test. Lower case letters are used for *T*. *distichum* saplings while upper case letters are used for *T*. *ascendens* saplings.

Mean DBH and tree height of bald cypress and pond cypress were significantly decreased along with submergence depth (Fig 4A and 4B). The crown area of bald cypress in MS was significantly larger than that in DS and SS, while DS and SS in bald cypress had no significant difference. In pond cypress, the crown area in MS and DS had no significant difference when compared to that in SS, respectively (Fig 4C). The primary branch number of bald cypress in MS and DS was significantly lower than that in SS, respectively, while no significant difference was demonstrated between MS and DS. In pond cypress, primary branch number was significantly decreased in DS, in contrast to no significant difference in MS, when compared to that in SS (Fig 4D). RDGR of both bald cypress and pond cypress were significantly decreased in DS as compared to that in SS, respectively. Furthermore, RHGR of the two species was significantly decreased with submergence depth. However, there was no significant difference to be detected in either RDGR or RHGR between bald cypress and pond cypress under any treatment (all *p* > 0.05), except for RDGR in DS (*p* < 0.05) (Table 3).

**Table 3:** Relative growth rates in DBH (RDGR) and height (RHGR) of *T*. *distichum* and *T*. *ascendens* saplings after experiencing three water level change cycles (means ± S.E., n = 9).

Index	Treatment^†^	*T*. *distichum*	*T*. *ascendens*
RDGR (mm mm^-1^ a^-1^)	SS	0.677 ± 0.034Aa^‡^	0.658 ± 0.028Aa
	MS	0.637 ± 0.028Aab	0.604 ± 0.028Aa
	DS	0.56 ± 0.025Ab	0.478 ± 0.029Bb
RHGR (m m^-1^ a^-1^)	SS	0.382 ± 0.01Aa	0.359 ± 0.009Aa
	MS	0.297 ± 0.007Ab	0.313 ± 0.007Ab
	DS	0.258 ± 0.011Ac	0.256 ± 0.01Ac

^†^Shallow submergence (SS), moderate submergence (MS) and deep submergence (DS).

^‡^According to Tukey’s test, the values with different letters are significantly different at the 0.05 level. Lower case letters represent the differences in RDGR and RHGR between the treatments within each species, whereas the upper case letters represent the differences between the two species under the same treatment.

Overall, there were effects for species, treatment and species × treatment (all *p* < 0.05) in DBH. In tree height, there was also an effect for treatment (*p* < 0.001), but no effects for species and species × treatment (both *p* > 0.05). Moreover, there were effects in crown area for species and treatment (both *p* < 0.001), but no effect for species × treatment (*p* > 0.05). There were effects in primary branch number for treatment (*p* < 0.001) and species × treatment (*p* < 0.05), in contrast to no effect for species (*p* > 0.05).

### Correlations

In bald cypress, there were significant correlations between *P*_*n*_ and growth indices. WUEi and Ls also exhibited a significant correlation with DBH and tree height in bald cypress. In pond cypress, only *g*_*s*_ significantly correlated with DBH and primary branch number (Table 4).

**Table 4:** Correlations between gas exchange parameters and growth indices for *T*. *distichum* and *T*. *ascendens* saplings (n = 27).

		DBH	Tree height	Crown area	Primary branch number
*T*. *distichum*	*P*_*n*_^‡^	0.622**^‡^	0.391*	–0.418*	0.335*
	*g*_s_	0.013	0.191	–0.191	0.311
	WUEi	0.517**	0.349*	–0.25	0.254
	Ls	0.525**	0.336*	–0.223	0.238
*T*. *ascendens*	*P*_*n*_	0.243	–0.113	0.02	–0.184
	*g*_s_	0.356*	0.12	0.27	–0.349*
	WUEi	–0.235	–0.311	–0.29	0.167
	Ls	–0.169	–0.242	–0.192	0.061

^†^Net photosynthetic rate (*P*_*n*_), stomatal conductance (*g*_s_), intrinsic water use efficiency (WUEi), limiting value of stomata (Ls) and diameter at breast height (DBH).

^‡^ *, *P* < 0.05

**, *P* < 0.01

## Discussion

### Pigment Response to Periodic Submergence

Environmental factors such as submergence and drought stresses could affect a plant’s leaf pigment composition and content [34–36], and thereby photosynthesis and the accumulation of assimilation products [37,38]. Typical symptoms of stress observed in tropical or temperate tree species subjected to soil submergence are leaf chlorosis and withering [39–41]. However, some flooding tolerant species could grow new leaves and adjust their pigment composition after flooding [42]. In our present study, bald cypress showed a parallel level in chlorophylls and carotenoid content, ratio of Chl a/b and ratio of Chls/Car, respectively, between DS and SS after three annual cycles of periodic submergence, indicating that deep submergence had no significant effect on pigment content of bald cypress most likely due to the enhanced ablity to remove active oxygen to repair submergence damage [13]. In our previous simulation study, first year bald cypress seedlings showed a significant decrease in chlorophylls and carotenoid content under flooding conditions [27]. Such a different response in photosynthetic pigment content in bald cypress in our present study might be due to the better tolerance abilities of the two-year-old bald cypress saplings to the periodic submergence. In addition, Li et al. [43] found that the total chlorophyll and Chl a/b of *Distylium chinense* raised to the control level after 60 days of recovery from flooding of 2 m. Qin et al. [44] also found that the total chlorophyll and Chl a/b in *Myricaria laxiflora* were kept at the same level as the control after 20 days of recovery from submergence above the plant top of 10 cm. In our present study, similar responses were also found in both bald cypress and pond cypress.

Pond cypress, however, showed the same level of chlorophylls and carotenoid content, ratio of Chl a/b and ratio of Chls/Car between submergence and control in our current study, suggesting that pond cypress could well adjust their pigment content so as to maintain their photosynthetic efficiency under these submergence conditions. In contrast, under simulated flooding conditions, chlorophyll content of pond cypress was significantly decreased [45]. The young seedling age (less than one year) in their study [45] may be responsible for such a difference. Belsky et al. [46] regarded this phenomenon as a positive response of plants after damage. This may be one of the main factors to consolidate pond cypress’ strong photosynthetic resilience to periodic flooding in the hydro-fluctuation zone of the TGDR.

### Photosynthesis Response to Submergence

In flood-tolerant species, the stomata can reopen [47–49] and photosynthesis can increase after flooding [50]. However, the ability of plants to recover from flooding varies as dependent on the stress intensity and duration, species as well as plant age [51,52]. In our current study, bald cypress saplings under MS and DS did not recover the *P*_*n*_ and *g*_*s*_ to the level of the SS, even though this species was widely understood to be hydrophilic. Thus, this result supported our first hypothesis that the photosynthesis of bald cypress will decline along with elevation drawdown due to longer duration of submergence stress. The lower *P*_*n*_ of bald cypress in MS may be attributed to lower stomatal conductance and chlorophylls and carotenoid content (Figs 2 and 3). Stomatal conductance can indirectly affect the leaf net photosynthetic rate through changing water potential [53] and leaf temperature, while directly inhibiting the leaf net photosynthetic rate by reducing the supply of CO_2_ to chloroplasts [54]. Under DS, as indicated by the lack of significant difference in *g*_*s*_, WUEi and Ls as compared to that of SS, respectively, the reduced *P*_*n*_ in DS may be caused by non-stomatal limitation, indicating that long-term deep submergence damaged the photosynthetic apparatus of bald cypress [55]. The recovery rate of plants after submergence may be explained by the amount of carbohydrate reserves [56]. This result is different to a previous finding that the *P*_*n*_ of bald cypress was not significantly affected by intermittent flooding [16]. Such different results might be due to the different experimental conditions, because in the latter study, an imposed flooding intensity of 2 cm above the soil surface with a duration of only two weeks under greenhouse growth conditions could not compare with the harsh conditions of the MS and DS set up in our current study. Other researches also found that flood tolerant species can recover their gas exchange rate under flooding, such as *T*. *distichum* [18,26], *T*. *ascendens* [21], or after water drainage, such as *Salix nigra* [27], *Nyssa aquatica* [57] and *Cynodon dactylon* [58]. Parad et al. [52] also found that the net photosynthesis and stomatal conductance of *Pyrus boissieriana* reached 74% and 50% of the control after 15 days of recovery, respectively. Thus, the fact that the net photosynthetic rate and stomatal conductance of bald cypress in this study did not recover to the control level after three years periodic submergence, may be mainly due to long-term severe stress or short-term recovery.

Pond cypress, on the contrary, resumed its *P*_*n*_ to control level under both moderate and deep submergence. This result corresponds with that in the study by Ismail and Noor [53], in which the photosynthetic rate of *Averrhoa carambola* seedlings subjected to soil flooding for 7 days attained similar levels of photosynthesis to those of controls 5 days after water drainage. Such a resumption in *P*_*n*_ indicated that pond cypress could recover well after a relatively long-term moderate and/or deep submergence. However, the recovery of *P*_*n*_ in pond cypress under both MS and DS may be caused by a higher amount of carbohydrate reserves [59] and enhancement of the ability to eliminate active oxygen [13]. In turn, recovery of *P*_*n*_ will benefit the plants by enabling storage of large amounts of carbohydrates to extend the maintenance of respiration and prolong their survival [60–62]. Thus, higher *P*_*n*_ would enhance the tolerance abilities of pond cypress for next submergence. In terms of the photosynthesis performance of bald cypress and pond cypress, pond cypress recovered better than bald cypress at lower elevation after submergence. These results support our thrird hypothesis, i.e. that pond cypress had a stronger photosynthetic resilience to long-term periodic deep submergence.

### Growth Response to Submergence

Plant growth and survival are closely related to resumption of their photosynthesis under flooding conditions which can contribute more energy and nutrient to the plant [63,64]. Survival rate is another critical indicator for flooding tolerance of plants [64, 65]. Bald cypress has flood-tolerance characteristics according to many previous studies [15,17,22–24]. Although studies about pond cypress are generally still less, it is also widely recognized as a flood-tolerant species [7,21,66]. In this study, no mortality was found in the two species during periodic submergence across consecutive three years, in accordance with the results of the simulation experiment conducted by Anderson and Pezeshki [18], indicating that both species have certain resistance abilities to submergence. Some field observations showed that flooding in the non-growing season had less harmful effects on plants [67]. When the flood season of the TGDR area changed from summer to winter, the metabolic rate and consumption of the plants may be reduced by the cold water in winter [68], thus possibly contributing to a relatively higher survival rate even though there was a long period of inundation [69,70]. Besides, the higher survival rate of the two species also related to the large amount of carbohydrate reserves [71].

Although many flood-tolerant species may shed their old leaves [61], elongate their stems [72] or reduce their biomass accumulation [73,74] when suffering from flooding, these traits are an important adaptive strategy for them to resist anaerobic conditions and retain vitality for growth after emergence from flooding. In the present research, the DBH and tree height of bald cypress decreased along with submergence depth, supporting our first hypothesis. However, Pezeshki and Anderson [16] found that the height of bald cypress under intermittent flood had no significant difference, while the basal diameter in intermittent flood was significantly larger, when compared to that of the control, respectively. Their short flooding time and shallow flooding condition [16] may contribute to these differences. Effler and Goyer [75] also found that flooding decreased the biomass of bald cypress. Colmer and Voesenek [76] documented that plants under long-term deep submergence will show traits of the Low Oxygen Quiescence Syndrome, slowing or ceasing their growth. Our present research found that there were significant correlations between *P*_*n*_ and growth indices in bald cypress (see Table 4), indicating that the lower DBH and tree height of bald cypress was mainly caused by lower photosynthesis. Moreover, a significant reduction in primary branch number might also contribute to a low accumulation of photosynthate, and thus result in such a reduced tree height and slow growth rate of bald cypress in both MS and DS as compared to that in SS. Although both moderate submergence and deep submergence had a comparable *P*_*n*_, they did not show a similar DBH in bald cypress. The reason might be that bald cypress under deep submergence needed to reallocate and consume more energy to resist the extreme environment than that under moderate submergence. Thus, our second hypothesis related to bald cypress was supported by these results.

The DBH and tree height of pond cypress also decreased along with submergence depth even though they had a higher *P*_*n*_ than bald cypress and a parallel *P*_*n*_ to the control. Therefore, these results did not support our second hypothesis in relation to pond cypress. Palta et al. [77] found that flood could affect annual productivity of *Taxodium* trees in the Savannah River floodplain. This phenomenon might be caused by greater energy expenditure as well as low productivity caused by lower crown area and primary branch number (Fig 4C and 4D). Extensive flooding is nearly a significant stress on forest survival and growth [31,78]. In this study, the crown area of pond cypress was smaller in three submergence treatments compared to that of bald cypress, even though they both started off at similar size. This may be explained by the leaf area of pond cypress being smaller than that of bald cypress, which decreased the total photosynthetic leaf area, thus leading to less accumulated assimilation product to support the branch expansion. *Vice versa*, the big crown area and primary branch number also resulted in a higher relative diameter growth rate of bald cypress under deep submergence.

In this study, bald cypress showed higher diameter growth rate in DS with lower net photosynthetic rate, while pond cypress showed lower growth rate with higher photosynthetic rate (Figs 2 and 4 and Table 3). Considering the sound growth of both species, these results indicated that both physiological and growth response should be investigated to screen the suitable plant species for vegetation restoration [79].

## Conclusions

The measured photosynthetic parameters and pigment content in bald cypress and pond cypress under three submergence treatments indicated that both species had various net photosynthetic rates and pigment contents under three submergence treatments after experiencing 3 years of water level changes. The DBH and tree height of the two species were decreased along with submergence depth (elevation drawdown). In conclusion, after three years of water level changes, the DBH and tree height of the two species greatly increased when compared to their baseline data, thus the two species appear to be plastic in response to long-term moderate and deep submergence in the hydro-fluctuation zone of the TGDR. Considering the higher photosynthetic resilience of pond cypress and higher plant growth abilities of bald cypress, both species can be applied in vegetation restoration of the riparian zone of the TGDR region.

In our field experiment, crown area of each sapling was larger than 1 m^2^ (Fig 4C), so overlapping crowns of the two species were observed due to higher planting density. Therefore, when implementing a similar restoration project in future, lower planting density should be considered for shelterbelt reforestation in the hydro-fluctuation zone of the TGDR or other large-dam reservoir areas.
